# Increased autumn and winter precipitation during the Last Glacial Maximum in the European Alps

**DOI:** 10.1038/s41467-021-22090-7

**Published:** 2021-03-23

**Authors:** C. Spötl, G. Koltai, A. H. Jarosch, H. Cheng

**Affiliations:** 1 Institute of Geology, University of Innsbruck, Innsbruck, Austria; 2 ThetaFrame Solutions, Kufstein, Austria; 3 Institute of Global Environmental Change, Xi’an Jiaotong University, Xi’an, China; 4 State Key Laboratory of Loess and Quaternary Geology, Institute of Earth Environment, Chinese Academy of Sciences, Xi’an, China; 5 Department of Earth Sciences, University of Minnesota, Minneapolis, MN USA

**Keywords:** Atmospheric dynamics, Cryospheric science, Palaeoclimate

## Abstract

The culmination of the glaciers in the European Alps during the Last Glacial Maximum (LGM) is one of the most intensively studied paleoglaciological events, but its trigger and forcing remain incompletely understood. Here, we provide evidence that the timing of this glacier maximum coincided within age uncertainties with a 3100 yr-long interval of subsurface warming (26.6 to 23.5 ka BP) as recorded by an archive preserved in caves, cryogenic carbonates. This interval of sustained permafrost degradation during one of the coldest intervals of the last glacial period calls for a fundamental change in the dry Arctic-style precipitation regime. Instead, heavy snowfall during autumn and early winter led to the accumulation of a seasonal snowpack insulating the ground from the winter chill. Combined with thermal modelling, the data provide compelling evidence that the LGM glacier advance in the Alps was fueled by intensive snowfall late in the year, likely sourced from the Mediterranean Sea.

## Abstract (web summary)

What controlled changes of glaciers in the European Alps at the time of their largest extent, about 25,000 years ago, is not well known. Here, the authors use cryogenic carbonates in caves to show that heavy snowfall during autumn and early winter was the main source of glacier growth.

## Introduction

The Last Glacial Maximum (LGM) is one of the best documented episodes in the Quaternary history of the Earth marked by a maximum in ice extent on the continent associated with a lowstand in global sea level. The LGM is stratigraphically defined to between 27.5 and 23.3 ka BP^1^ (ka, thousand years before 1950 AD), i.e., between the end of Greenland Interstadial (GI) 3 and the onset of GI 2.2^2^. Some authors prefer a slightly later onset of the LGM at 26.5 ka BP and an end at 19 ka BP^3^.

In the European Alps, the cradle of ice age research, the LGM marked the largest glacier extent of the last glacial cycle. Both the geometry of the ice-stream network and its large piedmont glaciers^4,5^ as well as the chronology of its buildup and decay^6–9^ have been thoroughly studied since many decades. Paleoglaciological studies have revealed that the ice divide during the LGM was located south of the modern weather divide, which suggests that prevailingly southerly circulation delivered Mediterranean-sourced moisture to the Alps at this time^10^. This pattern is in contrast to the modern advection of North Atlantic moisture from the (north)west and indicates that the southward displacement of the polar front caused mid-latitude cyclones to follow a more southerly route across the Mediterranean Sea, consistent with global circulation models showing evidence of a predominantly southerly flow pattern^11,12^. This scenario is corroborated by paleobotanical data showing a steep moisture gradient from the southern rim of the Alps (where forests survived locally during the LGM) to the northern foreland^13^. It is also consistent with paleoglacier models of the Alpine ice-stream network^14^, although one recent model study suggests a predominant moisture advection from the west^15^. Precisely dated evidence for a southward shift of the North Atlantic storm track during the LGM was reported from a cave system in Switzerland. Oxygen isotope data of stalagmites reveal a marked drop between 26.5 and 23.5 ka BP, coincident with the timing of the maximum ice volume in the Alps. The isotopic signal is consistent with moisture transport from the south across the main Alpine crest^16^. Although there is now compelling field- and proxy-based evidence that the maximum advance of Alpine glaciers during the LGM was triggered by precipitation associated with predominantly southerly moisture transport to and partly across the Alpine barrier^10–14,16^, it has remained unclear whether this “föhn-type” airflow and precipitation occurred seasonally or year-round.

Here, we show that permafrost in the unglaciated Obir region on the southeastern rim of the Alps warmed during the LGM, precisely at the time of the hypothesized southerly circulation. Subsurface warming during full stadial conditions requires a sufficiently thick seasonal snowpack to insulate the ground from the winter cold and therefore calls for a drastic change in the LGM hydroclimate. We argue that enhanced Mediterranean-sourced autumn to early winter snowfall provided the extra precipitation that allowed the Alpine glaciers to expand to their maximum extent for a few thousand years.

## Results

### Obir caves setting

**Fig. 1:**
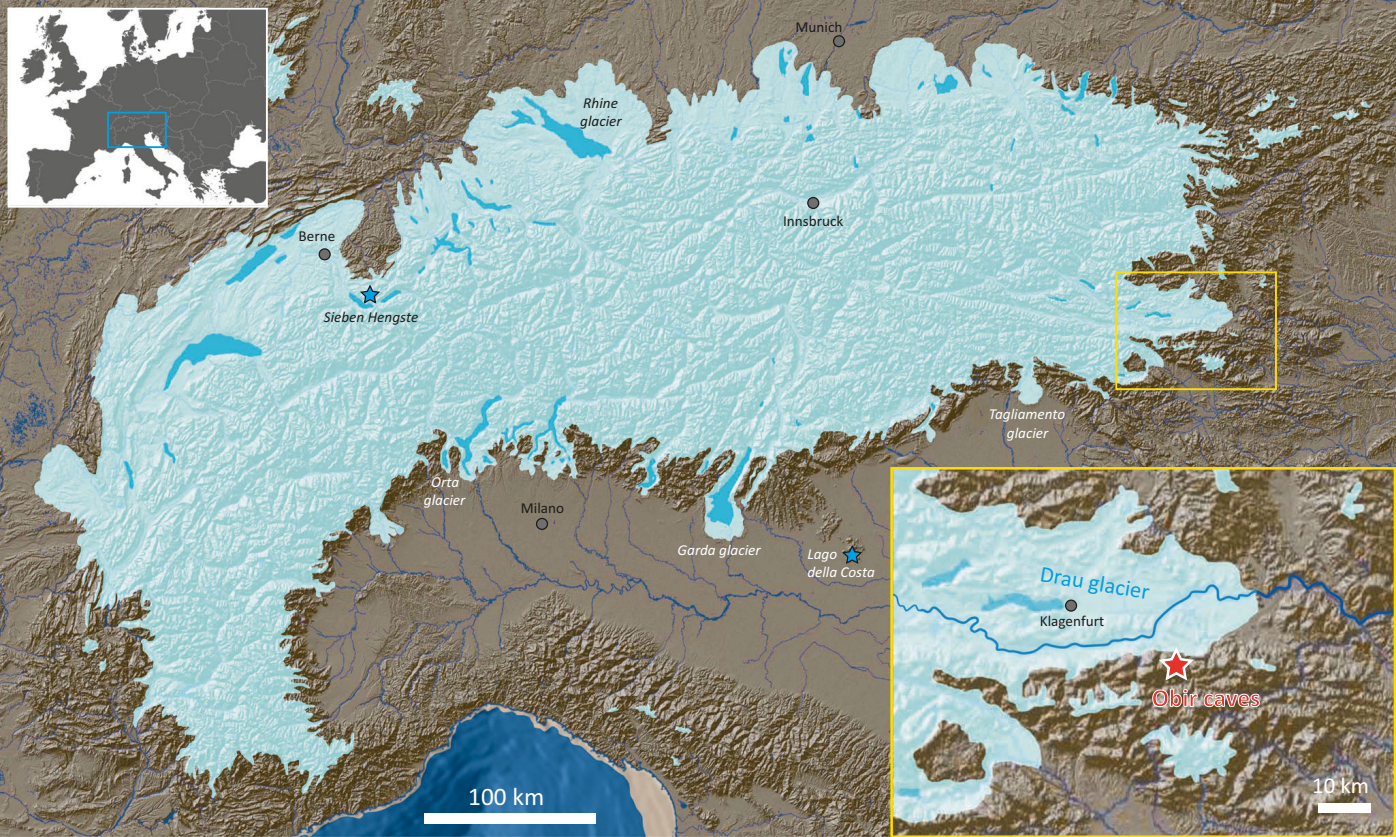
Relief map of the Alps showing the maximum extent of the Alpine Last Glacial Maximum ice-stream network. The study area of the Obir caves in southeastern Austria is enlarged (yellow rectangle). Other locations mentioned in the text are also labeled. Nunataks are omitted for clarity. Source of base map: © Geologische Bundesanstalt^64^.

Underground cavities on this eastern slope of Hochobir (2139 m a.s.l.) in the Northern Karawanks, collectively known as Obir caves (Fig. 1), were encountered during mining, which commenced in the 17th Century and came to an end during World War II. Most caves were discovered in the 1870s and 1880s. They are hypogene in origin, formed by aggressive, upwelling CO_2_-rich groundwater prior to or during the Neogene uplift of the mountain range^17^. The galleries, chambers, and pits form isolated caves, which can only be accessed by abandoned mining tunnels. The caves show abundant paleophreatic morphologies and lack evidence of later vadose entrenchment by cave streams. Breakdown blocks are wide-spread and none of the galleries extends to the surface except one narrow shaft that was artificially modified by mining activities. This study focusses on two parts of the Obir cave system located between ~1050 and 1100 m a.s.l., the 1.5 km-long Rasslsystem, and the adjacent but unconnected 0.9 km-long Banane system.

During the last glacial period, the area above the caves remained unglaciated. Only the summit of Hochobir hosted two small, steep, north-facing cirque glaciers^4^. During the LGM the southern margin of the Drau paleoglacier, whose tongue occupied the Klagenfurt basin, was only 2.5 km north of the study site^4^ (Fig. 1). The ice surface of the glacier tongue reached up to ~850 m a.s.l., ~250 m below the Obir caves.

Multi-annual monitoring shows that the air temperature in both cave systems is stable throughout the year (5.7–5.9 °C) and very close to the multi-annual mean of the air temperature outside the caves at this elevation^18^ (and unpublished data by the authors). In both caves, which lack perennial ice today, we identified coarse crystalline cryogenic cave carbonates (CCC for short). These carbonate speleothems form by very slow freezing of small water pools created by drip water on perennial cave ice deposits, providing robust ^230^Th-dated anchor points for thermal conditions very close to 0 °C^19^. The depths of the caves in which CCC were found range from 42 to 67 m below the surface (Supplementary Fig. 1).

### CCC distribution, composition, and age

CCC was identified in several chambers and passages in both caves (Supplementary Fig. 1), forming characteristic heap-like accumulations on the cave floor, up to 1–2 m wide, partly extending beneath breakdown blocks (Supplementary Figs. 2–5). Some of these accumulations show signs of later partial removal by drip water, and at two sites the crystals are partly cemented by a thin flowstone layer. Several morphological types of CCC occur both within the two caves and also within each accumulation. Most common are rhombic crystals as well as braided aggregates composed of spheres. Less common are complex rhombic crystal aggregates and spherulitic hemispheres (Supplementary Figs. 2–5). CCC accumulations are present in the same cave chambers where older stalagmites of mostly interglacial age have been conspicuously detached from their base along near-horizontal fractures. The lateral displacement is <~2 cm and the fractures are partly cemented by younger (Holocene) calcite.

The stable isotope data of CCC fall along broadly linear trends with similar negative slopes, and range from −4.5 to +5.4‰ in δ^13^C and from −21.7 to −8.6‰ in δ^18^O (Supplementary Fig. 6, Supplementary Data 1). These highly depleted O isotope and enriched C isotope values are consistent with precipitation from very slowly freezing water pockets enclosed in ice^19,20^. In contrast, Pleistocene and Holocene stalagmites from these caves show distinctly different values (Supplementary Fig. 6).

^230^Th ages were obtained from seven CCC accumulations, four from the Rasslsystem and three from the Banane system. The U concentrations vary between 1.4 and 4.3 ppm and are significantly higher than those of stalagmites from the same cave (on average ~0.1 ppm). Detrital contamination is low resulting in fairly accurate and precise ages ranging from 26.6 ± 0.2 ka to 23.5 ± 0.1 ka (Supplementary Data 2). For all but two of the accumulations, two different subsamples (in one case three) were analyzed. The results show that the ages within individual CCC accumulations differ between 165 and 1293 years (using the mean values). There is no relationship between the age of CCC and the depth of the corresponding cave chamber below the surface.

### Thermal modeling

**Fig. 2:**
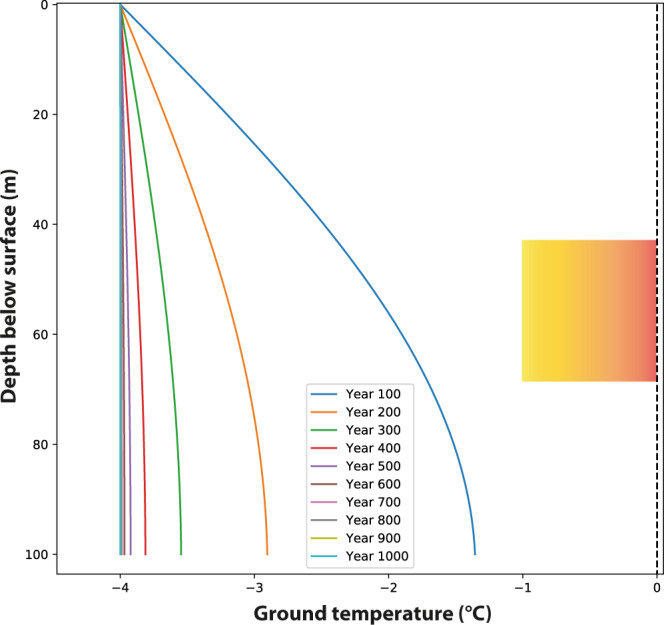
Scenario 1 simulating the development and deepening of permafrost during a cold and dry stadial. This model uses a mean annual air temperature of −4 °C and an initial temperature at the top of the ground of 1 °C. The curves depict the mean annual temperature. The rectangle with the orange shading marks the −1 to 0 °C window of possible cryogenic cave carbonates formation for the depth range of the Obir caves.

To quantitatively explore the relationship between thawing in the subsurface as recorded by CCC and climate change we applied a 1d heat-flow model (see Methods). In the first scenario, we simulated the initial development of permafrost as a result of a change towards a cold and dry stadial climate. As the starting condition, we assume a ground temperature of 1 °C (i.e., no permafrost) and used seasonal temperature values for stadial conditions consistent with regional proxy data (Supplementary Information). The results show that the atmospheric cooling propagates into the subsurface resulting in a permafrost zone some 80 m deep within 50 yr (Fig. 2, Supplementary Information).

**Fig. 3:**
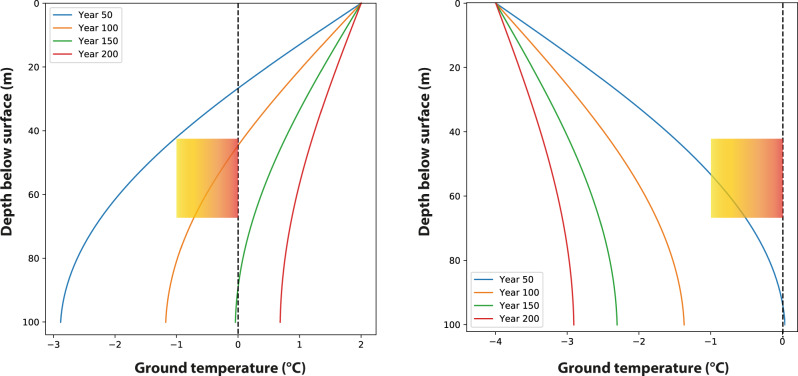
Scenario 2 simulating the degradation of permafrost during the transient warming associated with a 200 yr-long interstadial (left) and the thermal regime of the subsurface following the end of this warming (right). The mean annual air temperature at the ground surface is increased from −4 °C to 2 °C (left) and then reversed after 200 yr (right). The curves depict the mean annual temperature. The rectangles with the orange shading mark the −1 to 0 °C window of the possible formation of cryogenic cave carbonates for the depth range of the Obir caves.

The second scenario explores whether the warming of the subsurface recorded by the CCC could have been a delayed response to the GI 3, which commenced at 27.7 ka^2^, i.e. 1.1–4.2 ka prior to CCC formation, and lasted only some 200 years. As the initial setup we used the permafrost conditions simulated in scenario 1, i.e., the surface in near thermal equilibrium with the depth of the caves after ~1000 yr of stadial climate. The latter is a realistic figure given that the average duration of stadials in Marine Isotope Stages (MIS) 3 and 2 was at least 1000 yr^2^. We applied instantaneous atmospheric warming from −4 °C to 2 °C mean annual air temperature (MAAT) to allow the permafrost to thaw, consistent with the rapid warming at the onset of such interstadials as recorded in Greenland ice cores^21^. Allowing for the warming to last 200 yr, i.e., the duration of GI 3^2^, results in a temperature close to 1 °C at 70 m depth (Fig. 3, Supplementary Information). As soon as this interstadial warming comes to an end and cold stadial conditions return, however, permafrost quickly re-develops as in scenario 1 (Fig. 3).

**Fig. 4:**
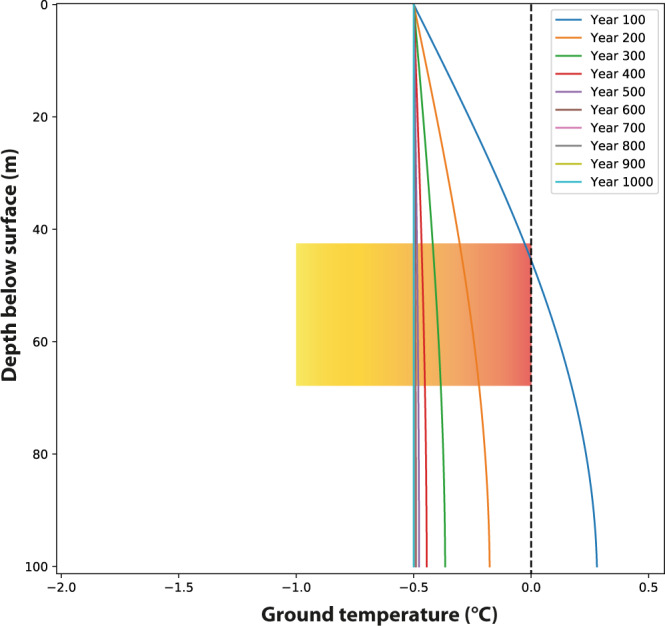
Scenario 3 simulating the impact of winter snow cover on the stability of the permafrost. This model uses the temperature profile at the end of a 200 yr-long interstadial (Fig. 3), a mean annual air temperature of −2 °C and a ΔT of 6 °C. The curves depict the mean annual temperature. The rectangle with the orange shading marks the −1 to 0 °C window of the possible formation of cryogenic cave carbonates for the depth range of the Obir caves.

The third experiment examines the effect of a seasonal snowpack on the thermal conditions in the subsurface (scenario 3). We used ΔT, the difference between ground temperature and air temperature, to describe the net thermal effect of snow cover on the ground temperature^22^. In the modern high Arctic, this parameter varies between −1 and 5 °C in summer, and between −10 °C and 20 °C during winter^22^. Although ΔT cannot be directly converted to seasonal or mean annual snow depth this parameter is strongly positively correlated with snow depth^23^. Studies in the Arctic of Russia have shown that an increase in snow thickness by 5–15 cm leads to a 1 °C increase in mean annual ground temperature and hence ΔT^22^. We kept the summer air temperature constant (as in scenario 2) but allowed for a small amount of winter warming (by 4 °C in January, from −15° to −11 °C) given that this scenario invokes a significant increase in autumn and early winter precipitation (i.e., MAAT −2 °C). We set ΔT at 6 °C, i.e., the snow cover attenuates the temperature at the top of the ground in January from −11 °C to −5 °C. This value of ΔT is within the range of modern ΔT observed on the North Slope of Alaska (4–9 °C; 23,24). We explored whether this scenario can explain prolonged permafrost thawing during the long GS 3 as recorded by CCC data. During the preceding GI 3 permafrost likely underwent some thawing and we used the thermal conditions at the end of this 200 yr-long interstadial (scenario 2) as the starting condition. The results show that the depth zone of the caves stays close to 0 °C for an extended period of time, e.g., the 50 m level reaches −0.1 °C after ~150 yr and then approaches thermal equilibrium at −0.5 °C (Fig. 4). Sensitivity tests using a range of ΔT values show that values between 4 and 7 are consistent with the CCC data (Supplementary Information).

For the three scenarios presented above, we also considered the thermal effects of ground ice, air advection, and latent heat transfer via seepage water. Although it is likely that they contributed to the rate of temperature change in the subsurface in response to climate change, we rule out that they significantly affected the magnitude of thermal changes as modeled with the conduction-only setup (Supplementary Information).

## Discussion

### Unique significance of CCC

CCC formation requires conditions very close to 0 °C, as shown by clumped isotope data^24,25^, and a delicate setup^19^. First of all, cave ice of sufficient thickness (typically >0.5 m) must be present. Second, the temperature of the ice and the chamber must be just slightly below 0 °C and the rock above the cave must be close to the melting point allowing the influx of (seasonal) drip water into the cave^26^. If the cave temperature is significantly lower than ~−1 °C the water refreezes rather quickly and results in massive deposition of ice either as ice stalactites and stalagmites and/or as thick-bedded floor ice^27^. If the dripping water encounters floor ice close to 0 °C, however, it will slowly melt the ice close to the impact point forming near-circular pools on the ice. Importantly, these depressions are filled with drip water that contains significant amounts of Ca and bicarbonate ions from the overlying karst rock (as opposed to very dilute ice meltwater). Third, the intra- and inter-annual temperature variations of the chamber and the ice therein must be very small (likely not exceeding a few tenths of a degree), thus allowing for a very slow downward re-freezing of the water in these pools. If the intra-annual temperature amplitude is larger and involves strong winter cooling then the re-freezing would be fast resulting in the precipitation of fine crystalline CCC varieties^19^. Only very slow freezing of these water pools and pockets in the ice will lead to a progressive increase in ionic concentrations and the eventual nucleation and slow growth of coarse crystalline CCC crystals and aggregates thereof.

CCC thus document that perennial ice close to the melting point was wide-spread in the Obir caves during the LGM. This is consistent with the occurrence of detached stalagmites in the same rooms and galleries, a deformation feature known from caves that were located in the permafrost zone during the last glacial period and where ice movement has caused wide-spread damage of older stalagmite generations^28,29^. Mapping of CCC and detached stalagmites, however, only provides a minimum estimate of the former extent of ice in these caves. The presence of broken stalagmites and stalactites in other rooms suggests that the ice extent may have been larger, but these features could also be unrelated to ice deformation.

Petrographic studies reveal the presence of only one type of CCC in some places and of two or three types in others, an observation also commonly made in other caves hosting CCC^29,30^. The ages of different CCC types within individual heaps commonly differ by up to several hundred years. There is a relationship neither between age and the stable isotopic composition nor between petrographic types and age. Close inspection of the C and O isotope data show that individual CCC accumulations fall along parallel linear trends (Supplementary Fig. 6). The slopes of these trend lines are similar and comparable to trends reported from other CCC sites in the Alps^30^ and other European caves^31^. These observations in conjunction with the petrographic data indicate that the water pools in the ice of Obir caves differed slightly in their stable isotopic composition and/or their isotope fractionation during freezing. This intra-cave heterogeneity is not surprising given the size of the studied caves, the number of CCC occurrences, and the time frame involved. Taken together, the 13 ^230^Th dates from seven CCC accumulations in these two caves document a time span of 3.1 ka (Supplementary Data 2) during which these caves contained ice accumulations very close to 0 °C.

### Paleothermal conditions of the subsurface

Caves in mountain ranges such as the Alps are typically well connected to the outside atmosphere and commonly show either strong, seasonally controlled ventilation (giving rise to an anomalously cold lower entrance) or trapping of cold air (in steeply descending or vertical single-entrance caves). Perennial ice accumulations in modern ice caves in the Alps are associated with these negative thermal anomalies. The mean annual air temperature in the ice-bearing parts of these caves is typically 3–4 °C lower than the MAAT at the same elevation outside^32^. Most modern ice caves in the Alps are, therefore, unrelated to discontinuous mountain permafrost, the lower boundary of which is presently located at ~2400 m on north-facing slopes and at ~3000 m on south-facing slopes in the Central Alps of Austria^33^.

The Obir caves are rather exceptional in this context and several lines of evidence suggest that their former ice accumulations reflect permafrost rather than a local thermal anomaly caused by airflow. First, owing to their hypogene origin, these caves were fairly isolated from the outside atmosphere prior to their discovery, strongly impeding air exchange with the atmosphere. Second, had there been forced airflow in these caves during the past (an unlikely scenario), the negative thermal anomaly would have been restricted to the area behind the lower (hypothetical) entrance, which is inconsistent with the distribution of CCC (Supplementary Fig. 1). Third, even today, after artificial adits have partially connected these isolated cavities, the interior of these caves (where the CCC were found) shows no evidence of thermal anomalies. The pre-mining subsurface thermal regime of the Obir caves was thus primarily governed by heat exchange with the surface via conduction. We rule out significant thermal effects owing to water entering the cave. Even during a warm and humid climate such as today, these caves lack streams. Only seepage water-feeding speleothems are present in parts of these caves. Significantly less infiltration is expected to have occurred during the cold conditions of glacial periods (Methods, Supplementary Information).

In conclusion, the Obir caves represent a rare example of mountain caves with very limited air exchange with the outside atmosphere (prior to their discovery) and lack thermal anomalies, thanks to their hypogene origin. This setting is key for drawing quantitative conclusions about the paleoclimate and past environments above the caves using the thermal and chronological information registered by CCC.

### Constraints from thermal modeling

Heat-flow modeling shows that the initial cooling of rock above the Obir caves is compatible with a stadial climate as suggested by proxy data. Essential for this subsurface cooling is the absence of a significant stable snowpack in winter, which leads to heat loss from the ground and a progressive deepening of the permafrost base. In the model, the Obir caves reach subzero temperatures within <50 yr of the onset of the cooling, depending on their depth, eventually approaching −4 °C after several hundred years (Fig. 2). This scenario is regarded as a good approximation of cold and arid stadials, e.g., Greenland Stadials (GS) 5.1, 4, and the early part of 3. This thermal regime is not suitable for CCC formation because the temperature of the subsurface is too low and the rock above the cave remains in the permafrost zone.

CCC record a warming of the permafrost regime starting at 26.6 ± 0.2 ka. This warming of the subsurface is not recorded by proxy data of any of the available regional LGM records^13,34^ (Supplementary Information) nor is there evidence for such a warming in records across Europe, even when taking some delay between the atmosphere and the subsurface into account. Modeling scenario 2 shows that this thawing of the permafrost cannot have been a delayed response to the atmospheric warming during GI 3, because both the start and end of this short thawing “window” were much too early compared with the dated CCC samples (Fig. 3). In contrast, these samples demonstrate that the thawing commenced 900 yr after the end GI 3 and lasted for 3100 yr. In addition, between ~26.5 and 23.4 ka BP, the climate in Europe became very cold as a result of Heinrich event 2^35–37^, intensifying subsurface cooling at Obir caves and closing the “CCC window” even earlier. We, therefore, reject the hypothesis that the short GI 3 was responsible for prolonged permafrost degradation and concomitant CCC formation thousands of years after this warming had ended abruptly. We regard it as likely, however, that this short interstadial contributed to the ice buildup in the caves by thawing the near-surface permafrost and allowing the meltwater to re-freeze in the cavities beneath which were still at subzero conditions for ~150 yr after the onset of the interstadial (Fig. 3). In addition, the more-humid interstadial climate likely gave rise to higher precipitation and hence infiltration, again promoting ice accumulation as long as the caves stayed well below 0 °C.

Scenario 3 shows that the cold climate of GS 3 and the CCC evidence can be reconciled by invoking a fundamental change in precipitation: very cold and dry autumns and winters with negligible snow cover gave way to slightly less cold and significantly more-humid autumns and winters characterized by a stable snow cover (Fig. 4). We hypothesize that this snow fell late in the year in order to effectively shield the ground from the chill of the long LGM winters. Where there is significant and stable snow cover in winter, the mean annual ground surface temperature is warmer than the MAAT^38,39^. In discontinuous and sporadic permafrost regions of the Arctic, the absence of seasonal snow cover is a key factor for permafrost development^22^. Our thermal modeling shows that adding a winter snowpack indeed leads to a warming of the permafrost. This subsurface warming is sensitive to both the winter temperature and snow depth (i.e., ΔT), but is consistent with the CCC timing and in particular with the extended period during which the depth range of the Obir caves remained close to 0 °C. This long-term stability close to the freezing point across a significant depth gradient (Fig. 4) also allowed some seepage water routes to stay open during LGM summers when the upper few meters of the ground warmed to several degrees C. Because also the rock beneath the active layer was close to 0 °C karst water did not readily re-freeze and could find its way into the caves creating drip water holes in the ice rather than contributing to the buildup of ice. This water contained Ca and bicarbonate ions and gave rise to CCC formation upon very slow re-freezing, e.g., during particularly cold intervals likely encompassing decadal time scales. We also evaluated the role cave ice formation/melting played during ground cooling/warming and found that the delay owing to the water-ice phase change is likely less than a few decades, thus not significantly affecting the overall thermal regime. This also applies to both air advection and water inflow. These processes play a substantial role in the heat exchange in many Alpine caves, but were likely of minor importance in the Obir caves owing to their hypogene origin and even less so during the LGM (Supplementary Information).

The CCC data, therefore, argue for fairly stable temperatures slightly below the freezing point in the Obir caves for the duration of GS 3. This paradox of a permafrost close to the thawing point during one of the coldest climate intervals of the last glacial cycle can be resolved by invoking a seasonal snowpack and hence a significant increase in solid precipitation. Looking at the fine-scale structure within GS 3, we observe a tendency of CCC to coincide with intervals of higher δ^18^O values in the Sieben Hengste stalagmite record (Supplementary Information). Although this relationship is blurred by the uncertainties associated with both data sets as well as delays between surface climate and the subsurface, the slow re-freezing events of the cave ice pools that led to the different CCC generations may have been in part triggered by small-scale variations in hydroclimate. The fine-scale pattern of CCC occurrences within GS 3, however, is certainly also related to local factors, including the localized and temporally changing presence of drip water in different parts of the two caves. In addition, there were certainly also galleries in these caves where no ice accumulated owing to the lack of water, hence CCC could not form there. Finally, post-LGM breakdown may have buried some of the CCC sites. Despite these confounding factors, the mapped CCC distribution and the spread of ages reflect a fairly stable subzero temperature regime for 3100 years recorded by individual CCC accumulations underneath former drips sites in places where floor ice was present.

### Paleoglaciological and paleoclimatic implications

Because CCC record conditions very close to the freezing point, the oldest date represents a minimum age for ice accumulation in a given cave. Perennial ice and permafrost conditions therefore already prevailed in the Obir caves prior to 26.6 ± 0.2 ka. Data on the distribution of permafrost during and prior to the LGM in the peri-Alpine realm are generally consistent with this observation. A study of rock glaciers in the Julian Alps south of the Karawanks identified the lowest rock glaciers at 1076 m a.s.l.^40^, close to the estimated equilibrium line altitude during the LGM in the Julian Prealps (1150 m^41^). An early numerical study in conjunction with paleoglaciological observations indicates up to ~150 m thick permafrost just outside the margin of LGM piedmont glacier lobes on the northern rim of the Alps^42^. And a regional compilation of permafrost features attributed to the LGM suggests that the southern limit of discontinuous permafrost straddled the southern rim of the Alps^43^.

**Fig. 5:**
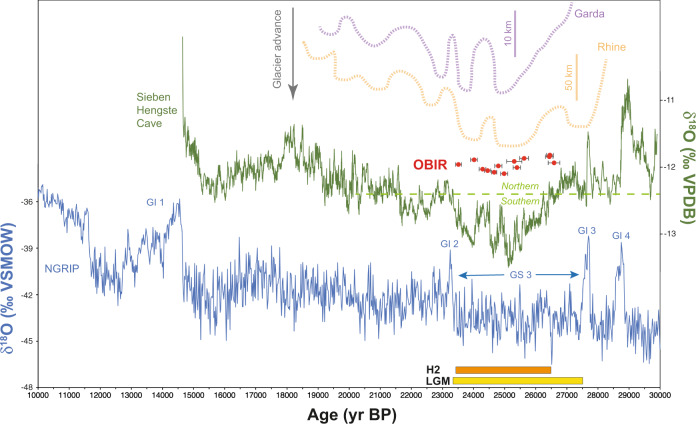
Ages of cryogenic cave carbonates from the Obir caves compared with key records for the Last Glacial Maximum. Cryogenic cave carbonates data (red, with two sigma error bars), oxygen isotope records from the North Greenland Ice Core Project (NGRIP^2^) and Sieben Hengste cave^16^ and the reconstructed extent of the Rhine and Garda paleoglaciers^9^. The green dashed line separates dominantly southern (i.e., Mediterranean-sourced) and northern/northwestern (i.e., Atlantic-derived) trajectories according to ref. ^16^. The yellow and orange bars at the bottom indicate the global Last Glacial Maximum (LGM)^1^ and the timing of Heinrich event (H) 2^35–37^, respectively. Selected Greenland Interstadials (GI) and Stadials (GS) are labeled. Note that the cryogenic cave carbonate data have no y-variability and that the offsets are only for visibility.

From 26.6 ± 0.2 ka onward the caves warmed to very close to 0 °C and CCC occurrences demonstrate that these conditions prevailed for the following 3.1 ka. This subsurface warming during one of the coldest intervals of the last glacial period is in contrast to regional proxy data but can be resolved by taking the presence of a snowpack in wintertime into consideration, calling for a fundamental change in the hydroclimate of the Alps. The onset of sustained subsurface warming in the study area was synchronous within dating uncertainties with a marked drop in oxygen isotope values of stalagmites from the Sieben Hengste cave in Switzerland (Fig. 5^16^). The following interval of low stalagmite oxygen isotope values, precisely constrained by ^230^Th ages, marks an interval of pronounced advection of southerly derived moisture to the Alps and matches the Obir CCC data both in terms of onset and duration (Fig. 5). This isotopic minimum was tentatively attributed to relatively warm and moist air advected to and across the Alps between spring and autumn^16^. This interpretation is only partially consistent with our data, because warm-season precipitation would have the opposite effect on the thermal regime in the subsurface. If falling as snow, it would insulate the ground from summer insolation. Conversely, lack of a snow cover fosters heat loss from the ground during winter, leading to a stabilization rather than a degradation of the permafrost (scenario 1). Our data support the hypothesis that the snow fell primarily before the main winter season, i.e., in autumn, in order to permit the buildup of a significantly thick insulating blanket against the chill of the long LGM winters. The timing of this hydroclimate switch is not only in excellent agreement with the Sieben Hengste record but also with less precise but spatially more comprehensive paleoglacier data sets from the Alps (Fig. 5). The LGM ice extent is best constrained chronologically by radiocarbon-dated organic remains in sediments of paleoglacier systems on the southern side of the Alps west of the Obir caves. The Garda piedmont glacier reached its frontal position just after 24.9 ka BP^9^, and the Tagliamento paleoglacier, which occupied a smaller catchment at lower elevation, reached its maximum extent somewhat earlier, between 26.5 and 23.0 ka BP^9^. Cosmogenic exposure data from the terminus of the Orta paleoglacier suggest a maximum extent between ~26 and 23 ka^44^. Data from glaciers flowing north, such as the Rhine paleoglacier, agree with data from the Southern Alps (Fig. 5) and confirm a glacial maximum at ~25–24 ka BP^6,7,45^. Some glaciers including Tagliamento show evidence for a second, smaller glacier advance centered at ~23–22 ka BP, followed by rapid down-wasting that commenced no later than ~22 ka^9,46^. The timing of the maximum ice extent around the Alps is thus in agreement within dating uncertainty with the duration of permafrost thawing at the Obir caves, lasting from 26.6 ± 0.2 to 23.5 ± 0.1 ka (Fig. 5), whereby the youngest CCC age represents a maximum age for the end of this thawing.

### GS 3—an anomalous stadial?

The Greenland ice core stratigraphy shows evidence of 26 stadials^2^, which resulted in a particularly cold and dry climate not only in central Greenland but also in central Europe^47–49^. Paleovegetation studies from southern Europe are consistent with a significantly cooler climate during stadials including GS 3, indicated by wide-spread steppe flora^50,51^. Mountain glaciers in the Mediterranean region, however, argue also for an increase in precipitation in order to account for the low equilibrium line altitudes^52–55^. General circulation models reveal a strong increase of the LGM cyclonic frequency during the winter half-year in Central Europe as part of a predominantly southerly and easterly flow pattern^11,12^. Depressions took a southerly route from the North Atlantic through the Mediterranean Sea due to the southerly displaced jet stream and polar front. Still, relatively warm surface waters in the Mediterranean Sea at the end of the glacial summer^56^ provided a major source of moisture, which gave rise to extensive glaciation in the Alps and other mountain ranges in and adjacent to the Mediterranean Sea. Our study provides evidence that this precipitation occurred predominantly in autumn and winter, which also today is the season when most heavy precipitation occurs along the southern fringe of the Alps, associated with the intense southerly flow against this mountain chain^57,58^.

Although not exceptional in terms of its duration, GS 3 stands out in the context of the last glacial period, because it occurred when the Laurentide ice sheet had reached its maximum extent^59^, largely coinciding with the global LGM sea level minimum. The Laurentide ice sheet had a major effect on the atmospheric circulation in the Northern Hemisphere and forced the jet stream over southern Europe and the Mediterranean Sea. Earlier stadials were associated with smaller extents of the Laurentide ice sheet^59^ and a concomitant more northerly route of the storm tracks across Europe. The southerly route of the storm tracks combined with a high cyclonic frequency during the winter half-year and very low temperatures were key elements in leading to the maximum expansion of the high-mountain cryosphere during LGM in the Alps and other mountain ranges close to the Mediterranean Sea.

## Methods

### Stable isotope analyses

We examined samples using a Keyence VHX-6000 digital microscope, and analyzed their stable C and O isotope composition. The latter was determined using a Gasbench II linked to a ThermoFisher Delta V Plus isotope ratio mass spectrometer. The results were calibrated against international standards and reported relative to the VPDB standard with a long-term precision of δ^13^C and δ^18^O of better than ±0.08‰ (1σ).

### ^230^Th dating

Selected crystals and crystal aggregates were used for dating, whereby 10–20 mg aliquots were drilled from these crystals under a laminar-flow hood. ^230^Th dating was performed at Xi’an Jiaotong University, China, using a ThermoFisher Neptune plus multi-collector inductively coupled plasma mass spectrometer. The method is described in ref. ^60^. Standard chemistry procedures were used to separate U and Th. A triple-spike (^229^Th–^233^U–^236^U) isotope dilution method was used to correct instrumental fractionation and to determine U and Th isotopic ratios and concentrations^60^. Uncertainties in U and Th isotopic measurements were calculated offline at the 2σ level, including corrections for blanks, multiplier dark noise, abundance sensitivity, and contents of the same nuclides in the spike solution. ^234^U and ^230^Th decay constants of ref. ^60^ were used. Corrected ^230^Th ages assume an initial ^230^Th/^232^Th atomic ratio of (4.4 ± 2.2) × 10^−6^, and the value for material at secular equilibrium with the bulk earth ^232^Th/^238^U value of 3.8. The correction is small and within the 2σ uncertainty range (Table 1). Final results are reported in years relative to 1950 AD (BP).

### Heat-flow modeling

We modeled the thermal conditions in the subsurface using a 1d heat-flow model, solving the heat equation utilizing finite differences as space discretizations alongside a forward Euler time-stepping scheme, stabilized with a diffusion-type Courant–Friedrichs–Lewy condition. The assumption of conductive heat transfer is justified given the fairly isolated nature of the Obir caves, which lack evidence of thermal anomalies common in caves showing forced ventilation or cold-trap behavior. The thermal regime of the shallow subsurface is governed by the interplay between the surface heat flow and the geothermal heat flow. The latter is comparably small (~50 mW/m^2^ in the Northern Karawanks^61^ and studies of deep karst systems have shown that it is greatly suppressed in the unsaturated zone owing to the combined effects of water inflow and air exchange^62^. Although the caverns of the Obir system are fairly isolated, given their hypogen origin, there is (and likely also was during the LGM) sufficient air exchange with the outside atmosphere to prevent the buildup of a geothermal gradient at the depth range of the caves (42–67 m). The cave system lacks streams and perennial water ingress points but shows evidence of seepage flow, leading to locally abundant dripstone formations. Although this very slow discharge likely only has a subordinate role in the heat exchange between the surface and the caves it is (and was during the LGM) very likely sufficient to eliminate the small contribution from the geothermal heat flow. We, therefore, modeled the thermal regime of the Obir caves without a geothermal heat-flow component. The thermal diffusivity of limestone ranges from about 1.1 × 10^−6^ m^2^/s to 1.2 × 10^−6^ m^2^/s at room temperature^63^. We used a value of 1.2 × 10^−6^ m^2^/s to account for some air-filled porosity in the Triassic limestone. The atmospheric temperature forcing was expressed by a sinusoidal seasonal cycle using the temperature of the warmest month (July) and of the coldest month (January) constrained by proxy data from published sources (Supplementary Information). Modeling results show the mean annual temperature against depth below the surface, but the program also calculates temperatures for the cold (January) and warmest month (July).

## Supplementary information

Supplementary Information

Descriptions of Additional Supplementary Files

Supplementary Data 1

Supplementary Data 2

## Data Availability

All data needed to evaluate the conclusions in the paper are present in the paper and/or the Supplementary Information and Supplementary Data 1 and 2.

## Code availability

The code of the 1D thermal model used in this work is available at 10.5281/zenodo.3767970 distributed under the GNU General Public License v3.0. A repository containing the model code can also be found at https://github.com/alexjarosch/diffusion_1D_temp/.
