# Supplementary material for: Increased autumn and winter precipitation during the Last Glacial Maximum in the European Alps
Source: Nat Commun. 2021 Mar 23;12:1839. doi: 10.1038/s41467-021-22090-7 (PMC7988052; doi:10.1038/s41467-021-22090-7)
Supplement: Supplementary file 1 — Supplementary Information [file 41467_2021_22090_MOESM1_ESM.pdf]

Supplementary Information

Increased autumn and winter precipitation during the Last Glacial Maximum in the European Alps

C. Spötl[1]*, G. Koltai[1], A.H. Jarosch[2], H. Cheng[3,4,5]

[1]Institute of Geology, University of Innsbruck, 6020 Innsbruck, Austria

[2]ThetaFrame Solutions, Hörfarterstrasse 14, 6330 Kufstein, Austria

[3]Institute of Global Environmental Change, Xi'an Jiaotong University, Xi'an, China

[4]State Key Laboratory of Loess and Quaternary Geology, Institute of Earth Environment, Chinese Academy of Sciences, Xi'an, China

[5]Department of Earth Sciences, University of Minnesota, Minneapolis, MN, USA

*corresponding author: christoph.spoetl@uibk.ac.at

CCC occurrences, petrography and stable isotopic composition

The Obir caves comprise a series of cavities between Hochobir and the deeply entrenched valley of the river Vellach. Parts of these caves have been opened to the public in 1991. Our study focusses on two caves outside the show cave, Rasslsystem and Banane.

A total of 11 individual spots of CCC were found in the Obir caves, 5 in Rasslsystem and 6 in the Banane system (Supplementary Fig. 1). All of these occurrences were sampled and studied petrographically as well as for their stable isotope composition. Seven of them yielded macroscopically and microscopically sufficiently clean crystals and were dated using the ^{230}Th method.

Supplementary Figs. 2 to 5 provide a documentation of each of these dated occurrences including their location in the cave, their macroscopic appearance and their dominant particle and crystal shapes.

Stable isotope analyses were performed on 0.1-0.2 mg aliquots of CCC crystals and aggregates thereof obtained using a hand-held dental drill. For larger particles such as hemispheres we also analyzed transects to investigate possible core-to-rim differences.

All carbon and oxygen isotope data fall into the compositional range previously reported for coarse crystalline CCC (*1*; Supplementary Fig. 6).

Paleotemperature constraints

Estimates based on the reconstructed LGM equilibrium line altitude in the Alps argue for a cooling of about 10°C relative to today (*2,3*). Chironomid data from Lago della Costa suggest July temperatures at the foothills of the Southern Alps about 10°C lower than in the 20th century (*4*). The latter values may be biased towards warmer temperatures because the foothills of the Southern Alps were glacial refugia for temperate tree species during the LGM (*5,6*). No reliable winter temperature proxies are available for the greater alpine realm.

Noble-gas data from regional aquifers suggest a LGM-Holocene difference of between 5-7°C north of the Alps and 8-9°C southeast of the Alps (*7*), broadly consistent with temperature estimates obtained from stable isotope data of such deep aquifers (*8*). Higher glacial-interglacial temperature differences are indicated by recent noble-gas and stable isotope data obtained from fluid inclusion in

stalagmites from Milandre Cave in the Swiss Jura Mountains (*9,10*). These data do not extend back to the LGM but indicate temperatures about 10°C colder than today for the Younger Dryas, which represents a minimum scenario for the LGM climate. Finally, climate models of the LGM in Europe capture a similar magnitude of temperature difference as the archives (*11-13*).

Based on the constraints from proxy data we used 10°C as a conservative estimate for the mean annual air temperature (MAAT) difference between the LGM and today. In our model we prescribe the seasonal cycle using the temperature of the warmest month (July) and of the coldest month (January) and used -15°C and 7°C, respectively, for the arid stadial scenario, resulting in a MAAT of -4°C. This is about 10°C lower than today's MAAT at the elevation of Obir caves. For comparison, today's mean January and July temperatures at Longyearbyen (Svalbard) are -14°C and 6°C, respectively (data source: https://de.climate-data.org).

Heat flow modelling sensitivity tests

(1) Arid stadial scenario (scenario 1)

We simulated the initial development of permafrost as a result of atmospheric cooling in a dry and cold stadial climate, starting with an unfrozen ground (+1°C) and dropping the MAAT to -4°C (January -15°C and July 7°C). The results (Fig. 2) show that the 0°C isotherm is progressively lowered, transforming the upper ca. 70 m of rock (where the caves are located) into a permafrozen zone within less 50 yr. We evaluated the sensitivity of this cooling scenario first by varying the value for the initial ground temperature. Using a +2 (+3)°C value results in a delay of the subsurface cooling. It takes about 50 (60) yr for the 0°C isotherm to reach a depth of 60 m. Second, we varied the MAAT by ±1°C (from -5 to -3°C) by adjusting the mean January and July values and leaving the initial ground temperature at 1°C. The results show that a lower (higher) MAAT of the modelled stadial results in equivalent temperatures in the subsurface, whereby the upper ca. 70 m of rock become permafrozen within ca. 35 (45) yr.

(2) Interstadial warming scenario (scenario 2)

In the second scenario we examined the thermal changes in the subsurface triggered by a short interstadial, such as GI 3 or 4, which lasted for only about 200 yr each (*14*). Very few proxy records are available for these short-lived interstadials; none from within the Alps because paleovegetation records within (and also north of) the Alps terminated at about 30 ka BP (*15,16*) and Central Alpine glaciers started to advance into the large inneralpine valleys at the MIS 3/2 transition (*17,18*). The only records partly covering this time period are from the foothills of the Southern Alps, a climatically favored region and one of the northernmost refugia of thermophilous trees in Europe during the LGM (*5*). Chironomid data suggest July air temperatures at Lago della Costa ranging from about 11-12°C to 15-16°C (*4*), i.e. a drop in July temperature between about 11-12°C and 7-8°C compared to today (23°C). Taking this drop in July temperature and the higher elevation of the Obir caves compared to Lago della Costa (ca. 1100 m versus 7 m a.s.l.) into account we used a mean July air temperature of 12°C and a mean January temperature of -8°C for GI 3 in the study area. The resulting MAAT (+2°C) is 6°C higher than that of the preceding stadial.

We evaluated the sensitivity of this warming scenario by varying the MAAT between 0.5°C (minimum value required to melt the permafrost) and +2.5°C, which we regard as a maximum estimate for this weak interstadial (i.e. only 2.5°C colder than today). The results show that a MAAT of 0.5°C (using mean January and July temperatures of -9° and 10°C, respectively) is insufficient to warm the depth range of the caves to 0°C within 200 yr, i.e. the duration of GI 3. In contrast, the caves warm to 0°C within about 70-120 yr using 2.5°C MAAT. Allowing the warming to last for up to 200 yr results in a temperature of 1.2°C at 70 m depth.

These simulations show that the transient warming of the subsurface as a result of an interstadial warming of transects the thermal "CCC window" only during rather warm scenarios and the warming is too short to explain the range of CCC ages which are between 0.9 and 4.0 kyr younger than the end of GI 3.

(3) Snow-rich stadial scenario (scenario 3)

We tested the sensitivity of this setting in two ways. First, we considered ΔT which describes the attenuation of the winter cold by a snowpack. Values <4°C, representative of a rather thin winter snowpack, are insufficient to warm the ground to close to 0°C and the depth interval <60 m remains in the permafrost zone. On the other hand, at ΔT >7°C, the ground temperature at the depth interval of CCC formation continuously stays ≥0°C, which would lead to a disappearance of cave ice, inconsistent with the documented duration of CCC precipitation. In addition, ΔT >7°C result in an unrealistically high buffering of the winter temperature at the top of the ground.

Second, we considered the winter temperature. The lower this parameter the higher ΔT must be for the caves to reach and stay in the "CCC window". Using the same starting conditions (i.e. output of scenario 2) even a ΔT of 8°C is insufficient to reach these near-zero conditions if January temperatures are lowered from -11° to -15°C.

Comparison of CCC and Sieben Hengste stalagmites

The ^{230}Th dates span a 3100 yr-long interval during which CCC formed in different parts of the caves (26.6 to 23.5 ka BP). Within this time period there is no obvious pattern and some ages are less precise than others allowing for small shifts in time (within age uncertainties). In order to explore whether the distribution of CCC ages may reflect a hydroclimatic signal we compared them to the oxygen isotope record from Sieben Hengste Cave in the Western Alps, currently the only continuous high-resolution proxy record in the greater Alpine realm for the LGM, also based on high-precision ^{230}Th dates (*19*). The $\delta^{18}O$ values of these stalagmites record a precipitation and a temperature signal (*19*). We obtained time-averaged $\delta^{18}O$ values from the Sieben Hengste record for each CCC sample with an averaging window based on each CCC date plus/minus the respective 2σ age uncertainty. These average $\delta^{18}O$ values should represent the hydroclimatic conditions during which each CCC sample formed. In addition, we investigated possible delays between the $\delta^{18}O$ record and CCC formation. Two types of delays were considered: (a) a delay of the atmospheric signal recorded in the Sieben Hengste speleothems. The average transfer (residence) time of the drip water that fed these stalagmites is unknown but was likely in the range between a few years up to a few decades, taking into consideration the depth of the cave some 250 m below the surface and its superposition by a temperate glacier (*19*). (b) Delays associated with the heat transfer from the surface into Obir caves. Part of the latter delay is cancelled out already by the delay recorded by the Sieben Hengste data. Supplementary Fig. 7 shows that although there is not a clear-cut relationship between the two data sets, CCC in Obir caves tend to coincide with intervals of higher $\delta^{18}O$ values of Sieben Hengste stalagmites. Following the interpretation of (*19*) that less negative $\delta^{18}O$ values indicate slightly higher temperatures and less precipitation (from the South), this pattern could imply that CCC formed preferentially during such discrete intervals, which lasted between a several decades and a couple of centuries. Given that the cave temperature likely did not fluctuate by more than a few tenths of a degree, this could mean that the episodes of slow re-freezing of cave ice pools and subsequent growth of CCC during GS 3 may have been partially triggered by small-scale variations in the hydroclimate. Nevertheless, the observation that the different CCC generations are spread over two cave systems illustrates that heterogeneities in the caves (e.g., presence of cave ice, presence of drips) certainly also played an important role.

Thermal impact of ground ice

For the three scenarios presented in this study pre-existing ice in the caves has the potential to prolong the response time calculated by the thermal model. In case of ground warming, where thermal energy enters the system, a phase change from solid to liquid absorbs thermal energy (i.e., enthalpy of fusion) and counteracts the warming process. In case of ground cooling, where thermal energy leaves the system, a phase change from liquid to solid releases thermal energy (given by the enthalpy of solidification) and counteracts the cooling process. As both, enthalpy of fusion and solidification, are equal, a potential time delay can be estimated, assuming a 1-2 m thick ice body and the range of modelled thermal gradients when the ground temperature passes 0°C, which ranges between 10 and 20 years.

Hence the melting of an ice body in the caves (the accumulation of cave ice) could possibly prolong the ground warming (cooling) by up to 20 years.

Thermal impact of air advection

While forced air advection can have a significant impact on the thermal regime of the near-entrance zones of caves with multiple openings at different elevations, this does not apply to Obir caves. Their geometry and configuration argue for a very restricted air flow regime prior to the mining activities which resulted in artificial connections of previously rather isolated caves. As a consequence, air exchange between the caves and the outside atmosphere was also likely highly restricted during the LGM but likely sufficient to suppress the weak geothermal heat flow. The qualitative effect of weak air advection on the thermal regime of the subsurface would be an acceleration of transient temperature changes, e.g. during climate transitions, without affecting the magnitude of these changes.

Thermal impact of water inflow

Water flowing through permeable rock has a major influence on the thermal structure of the latter, essentially – in settings such as mountain caves – cooling the subsurface and suppressing the local geothermal gradient (*20-22*). Although we did not model the thermal impact of water in the Obir caves, two lines of observations argue that water infiltration during the LGM was very low and its impact on the thermal structure of the karst rock was accordingly small: (i) Obir caves lack cave streams and high-discharge water ingress points. Water entering the caves today is largely seepage that slowly finds its way through the host rock to the caves, picking up ions and leading to the formation of warm-climate stalactites, stalagmites and flowstones due to degassing of carbon dioxide upon entering the cave chambers. This water is thermally equilibrated with the host rock and the fluxes involved are much smaller than in (non-hypogene) karst systems characterized by large conduits. (ii) The climate during the LGM, even when considering an increase in late-season precipitation, was significantly less humid than today, strongly limiting the amount of water available for infiltration. We also rule out influx of glacial meltwater into the caves because there were no glaciers in the catchment of the cave. Discharge from the two small cirque glaciers which were present on the Hochobir summit during the LGM (*23*) did not reach the caves because there is a mountain ridge in between. Qualitatively speaking, the net effect of any type of water infiltration would have been an acceleration of transient temperature changes, analogue to air advection, i.e. no significant change in the amplitude, but a shortening of the reaction time of climate changes propagated into the subsurface.

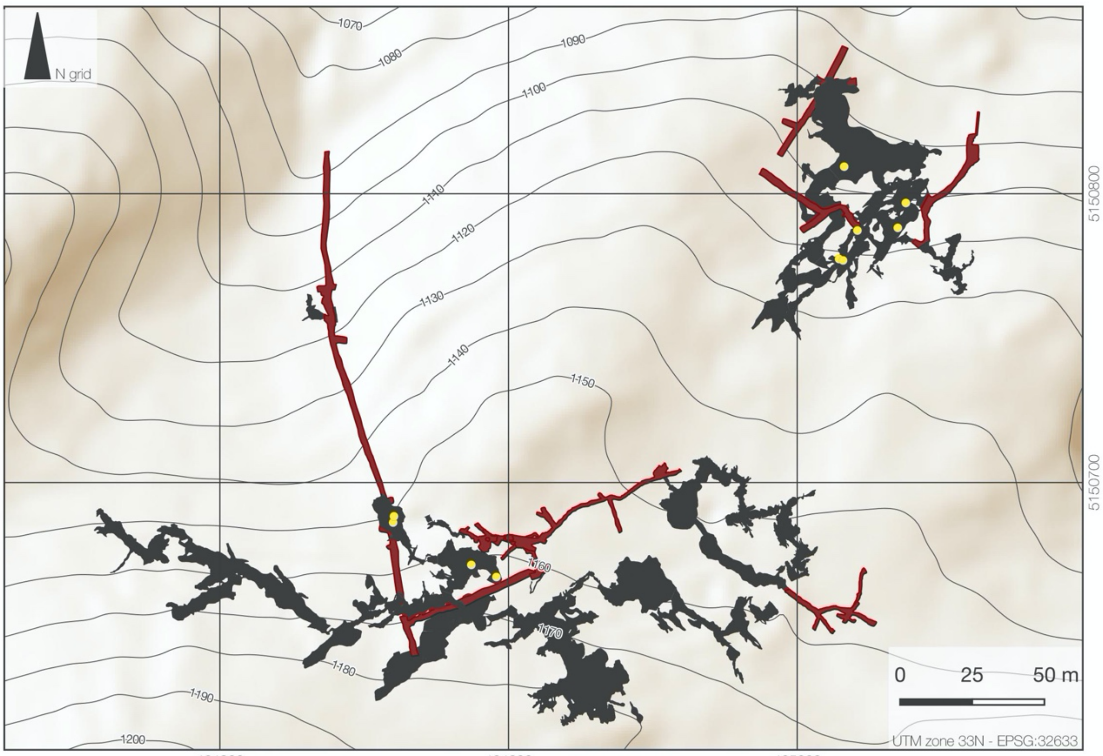

Suppl. Fig. 1. Map of Obir caves and occurrences of CCC. Digital elevation model with 10 m contour lines superimposed on the cave survey of the two studied caves shown in dark gray, Rasslsystem in the south and Banane system in the northeast. Some of the mining adits that exist in this area are shown in dark red. CCC occurrences are marked by yellow dots.

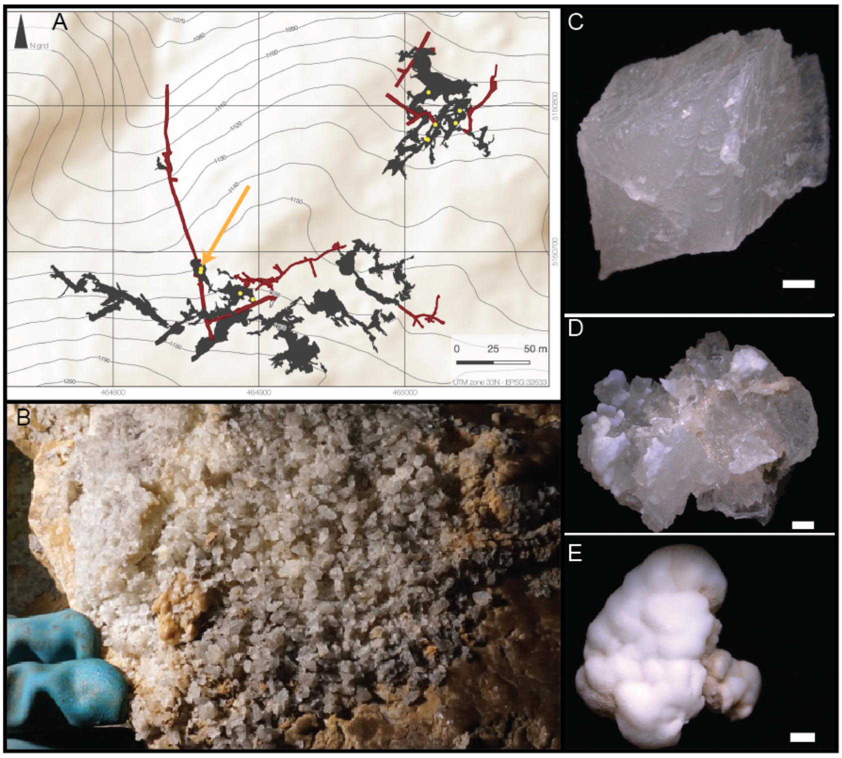

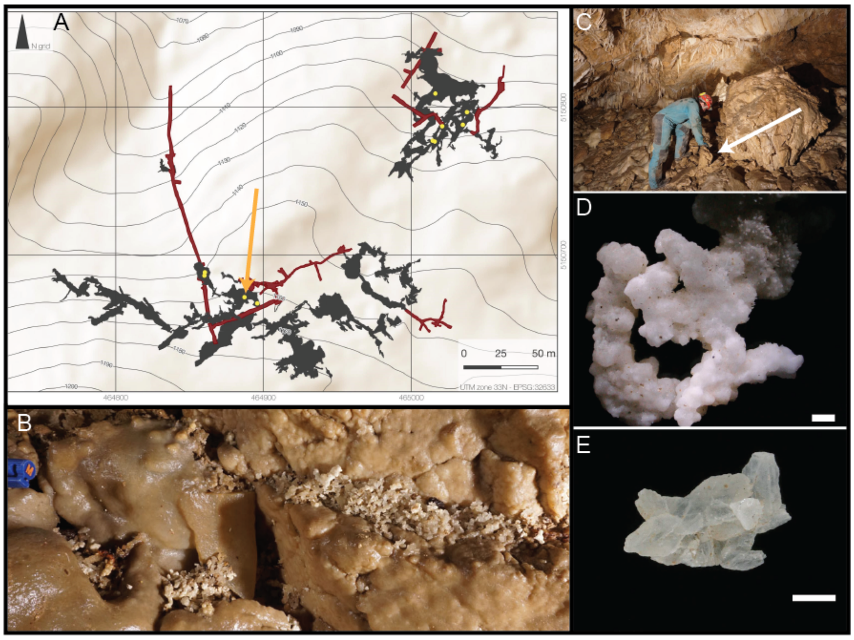

Suppl. Fig. 2. CCC sites Obi 97 (top) and Obi 106 (bottom). Top: Loose and partially cemented CCC covering 1-2 m^2 in Sendesaal, a fairly isolated chamber of the Rasslsystem (A-B; fingers of glove for scale in B). CCC are mostly translucent and show rhombic (C) and split crystal structure and are variable in size, up to 1.2 cm (E-F). Some of the CCC are coated by a younger white calcite layer that is of the same age. Some of the CCC were cemented and were not sampled. **Bottom**: This spot was found in the center of Pseudosaal (A) and consists of an assemblage of loose globular CCC, partly beneath a breakdown block covering an area of about 0.5 m^2 (B-C; tip of blue pen for scale in B). CCC occur mostly as aggregates up to 0.5 cm size comprising globular (D) and rhombohedral crystals (E). Globular aggregates are most abundant at this site. The site shows some indications of removal of CCC by drip water. White scale bars 1 mm.

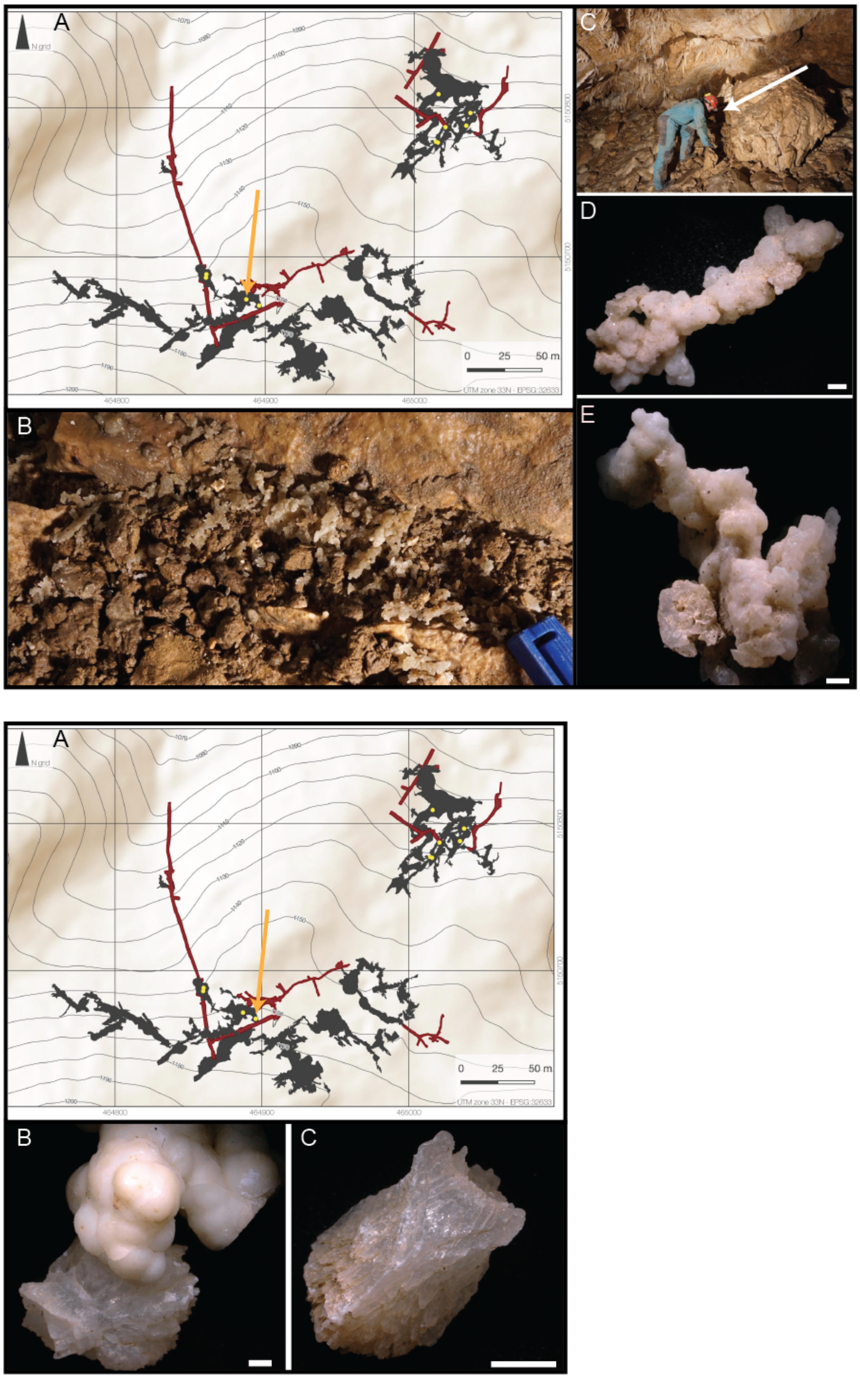

Suppl. Fig. 3. CCC sites Obi 107 (top) and Obi 108 (bottom). Top: Loose white and partly translucent globular CCC (D-E) found approximately 60 cm from site Obi 106, beneath the same breakdown block (A-C; tip of blue pen for scale in B). Field observations and ^{230}Th ages indicate that Obi 107 is a spot separate from Obi 106. CCC cover an area of about 0.5 m^2 and the largest aggregates reach 1.5 cm (D). **Bottom**: Translucent and white CCC were found as individual crystals and aggregates approximately 20 m from Obi 107 in a small chamber connected to Pseudosaal via a narrow passage. Translucent crystals of rhombic structures are up to 9 mm in size, while crystal aggregates are up to 1.6 cm. As only few scattered CCC were found there it is likely that some were washed away and disappeared between breakdown blocks due to dripping water. White scale bars 1 mm.

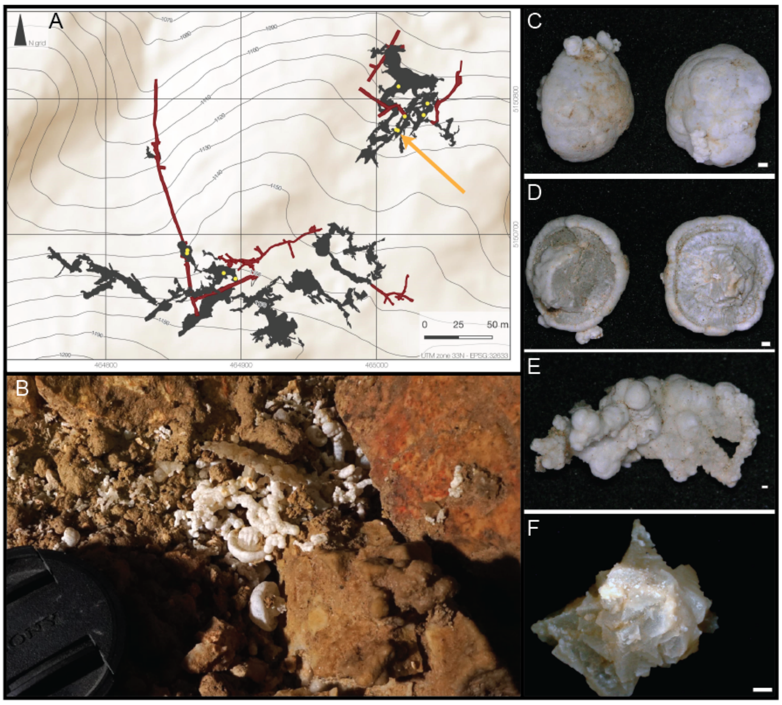

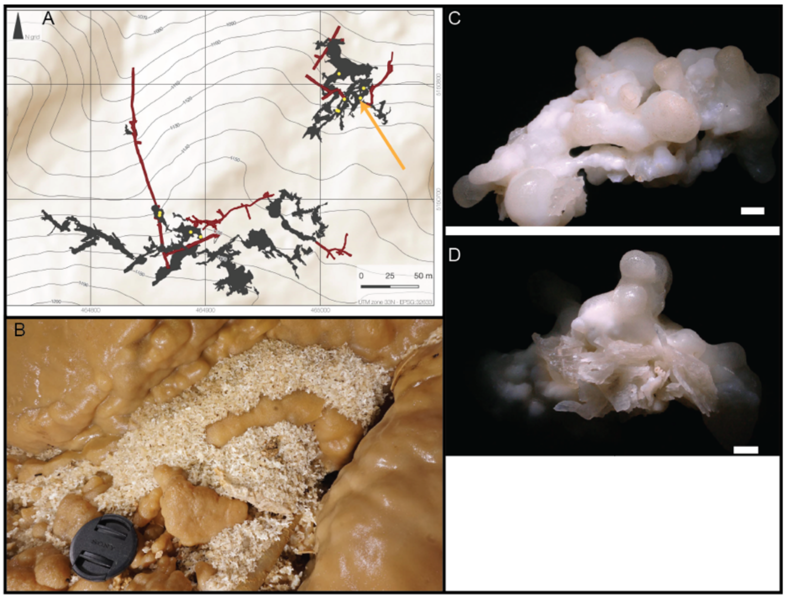

Suppl. Fig. 4. CCC sites Obi 110 (top) and Obi 111 (bottom). Top: CCC on the floor of an ascending passage near Sandgang in Banane system present as loose hemispheres, rhombohedral and skeletal-globular multi aggregates covering an area of about 1.5 m^2 (A-B; camera lens cap for scale in B). Most abundant morphologies are translucent hemispheres with a root, resembling a mushroom (C-D). These particles are 1.0-1.5 cm in size and are often coated by a mm-thick white calcite layer of cryogenic origin (based on stable isotope data). Multi-aggregates (E) are larger, often 4.0-4.5 cm in length. Rhombohedral aggregates (F) are present but rare. **Bottom**: Translucent skeletal crystals, hemispheres and skeletal-globular multi-aggregates of several mm in size were found mostly cemented to the floor in a terminal chamber of the Banane system. CCC cover an area of 2-3 m^2 showing multiple heaps with clear signs of partial removal due to dripping water (A-B; camera lens cap for scale in B). Several sheared and broken stalagmites are present in the same chamber indicative of the former presence of a perennial ice body. Skeletal crystals and hemispheres are less than 0.8 mm in length, while multi-aggregates reach 1.7 cm in size. White scale bars 1 mm.

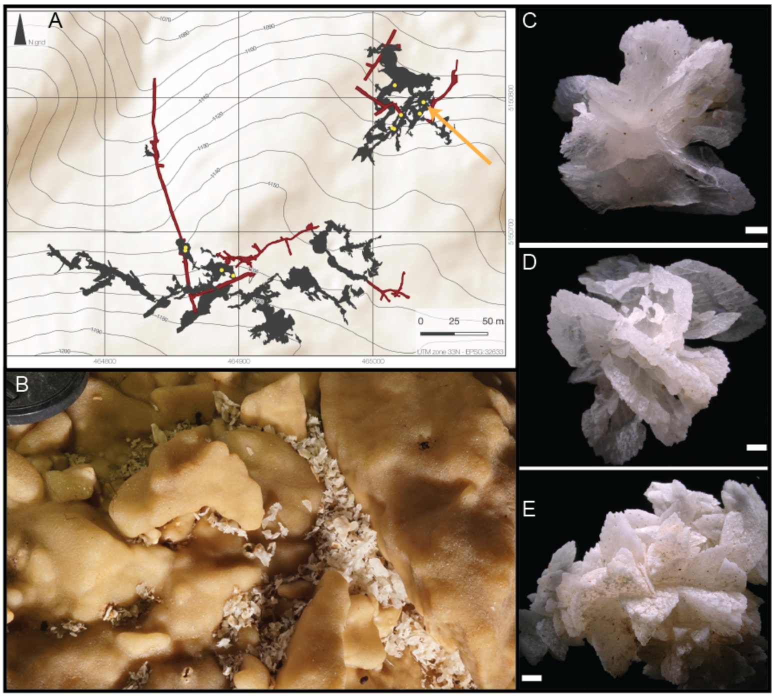

Suppl. Fig. 5. CCC site Obi 113. Loose skeletal, split and rhombic crystal aggregates occur on the floor of cave passage leading from the terminal chamber (Obi 111) to the central parts of the Banane system (A-B; camera lens cap for scale in B). No evidence of a younger calcite cementation was observed. Several broken stalagmites and one sheared stalagmite are present in close vicinity of this site. Crystal aggregates are translucent and commonly 1 cm in size, but slightly larger aggregates were also observed and occupy an area of about 0.5 m^2. White scale bars 1 mm.

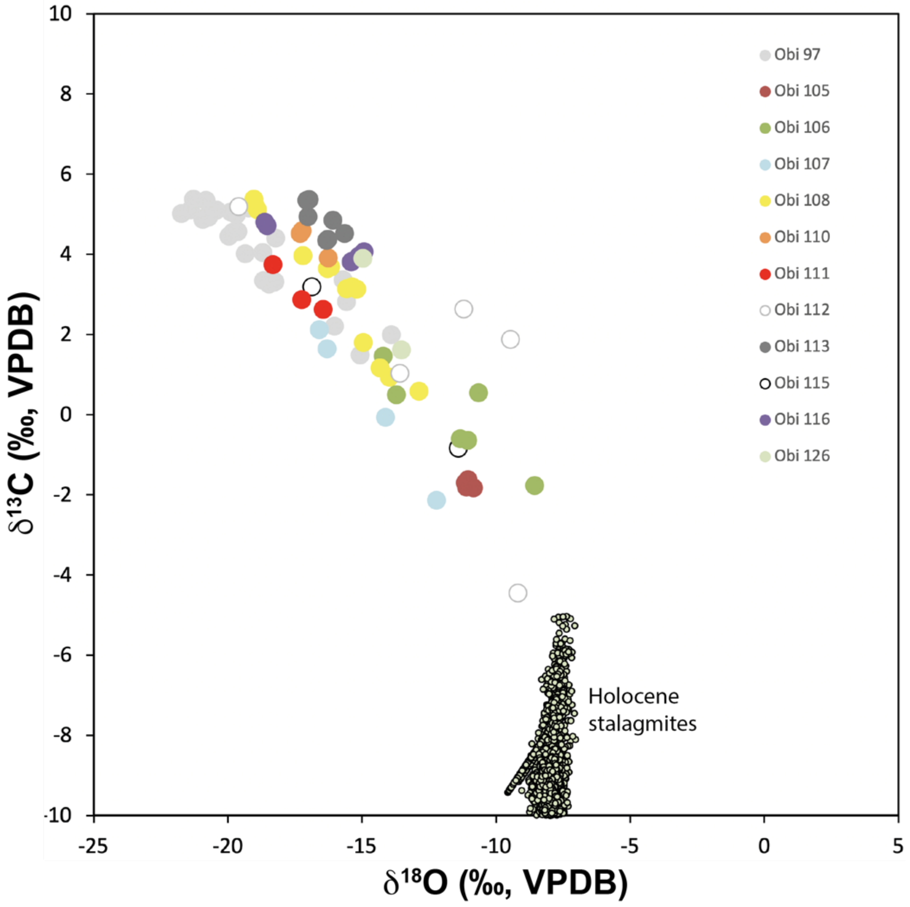

Suppl. Fig. 6. Stable isotopic composition of CCC from the Obir caves. Samples from different CCC sites are color-coded (see Supplementary Figs. 2 to 5) and data of Holocene stalagmites from this cave (C. Spötl, unpublished data) are shown for comparison.

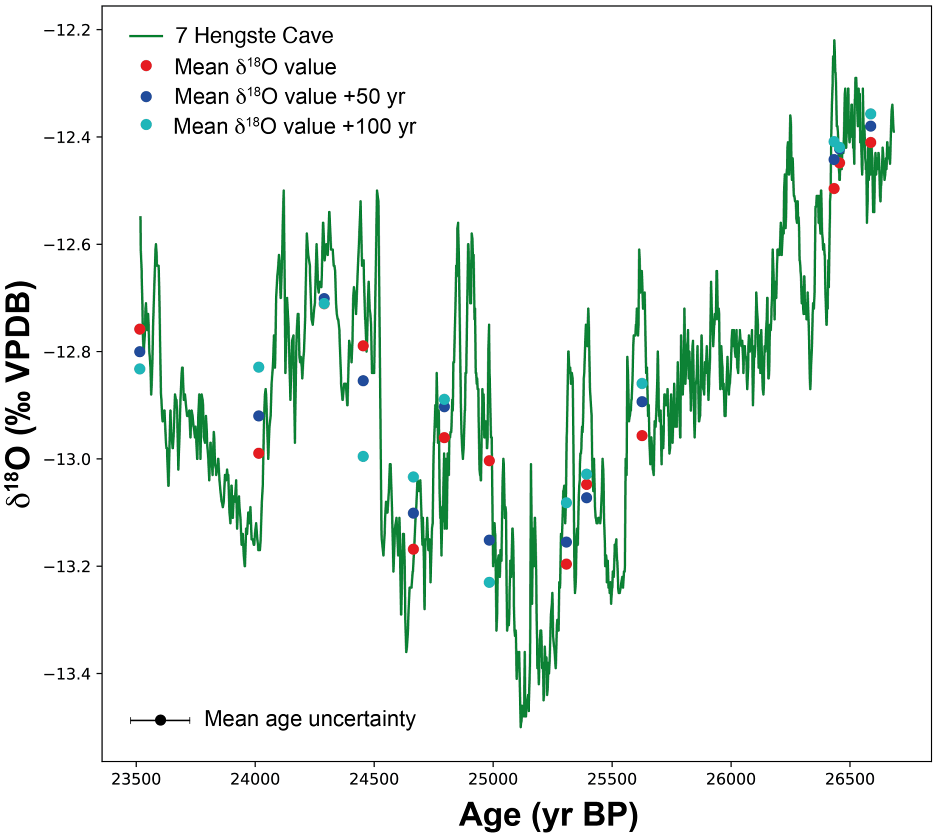

Suppl. Fig. 7. Comparison of CCC ages from Obir caves and the oxygen isotope record of stalagmites from Sieben Hengste Cave, Switzerland. Colored dots display time-averaged $\delta^{18}O$ values of the high-resolution speleothem record from Sieben Hengste Cave (*19*) for intervals when individual CCC samples formed in the Obir caves, and for different delay times. Averaging windows are based on the ^{230}Th ages of individual CCC samples including their 2σ age uncertainties. Red dots have no time delay. For the blue and light blue dots, the averaging window has been moved into the past by 50 and 100 years, respectively, to assess possible delays between the $\delta^{18}O$ signal and CCC formation.

Supplementary References

1. Žák, K., Onac, B.P., Kadebskaya, O., Filippi, M., Dublyansky, Y. & Luetscher, M. Cryogenic mineral formation in caves. In: *Ice Caves* (eds. A. Perşoiu & Lauritzen, S.-E.), 123–162 (Elsevier, Amsterdam) (2018).
2. Haeberli, W., Rellstab, W. & Harrison, W.D. Geothermal effects of 18 ka ice conditions in the Swiss Plateau. *Ann. Glaciol.* **5**, 56–60 (1984).
3. Kuhlemann, J., Rohling, E.J., Kubik, P., Ivy-Ochs, S. & Kucera, M. Regional synthesis of Mediterranean atmospheric circulation during the Last Glacial Maximum. *Science* **321**, 1338–1340 (2008).
4. Samartin, S., Heiri, O., Kaltenrieder, P., Kühl, N. & Tinner, W. Reconstruction of full glacial environments and summer temperatures from Lago della Costa, a refugial site in Northern Italy. *Quat. Sci. Rev.* **143**, 107–119 (2016).
5. Kaltenrieder, P. et al. Environmental and climatic conditions at a potential Glacial refugial site of tree species near the Southern Alpine glaciers. New insights from multiproxy sedimentary studies at Lago della Costa (Euganean Hills, Northeastern Italy). *Quat. Sci. Rev.* **28**, 2647–2662 (2009).
6. Monegato, G. et al. Sedimentary evolution and persistence of open forests between the south-eastern Alpine fringe and the Northern Dinarides during the Last Glacial Maximum. *Palaeogeogr. Palaeoclimat. Palaeoecol.* **436**, 23–40 (2015).

7. Corcho Alvarado, J.A., Leuenberger, M., Kipfer, R., Paces, T. & Purtschert, R. Reconstruction of past climate conditions over central Europe from groundwater data. *Quat. Sci. Rev.* **30**, 3423–3429 (2011).
8. Jiráková, H., Huneau, F., Celle-Jeanton, H., Hrkal, Z. & Le Coustumer, P. Insights into palaeorecharge conditions for European deep aquifers. *Hydrogeol. J.* **19**, 1545–1562 (2011).
9. Ghadiri, E. et al. Noble gas based temperature reconstruction on a Swiss stalagmite from the last glacial–interglacial transition and its comparison with other climate records. *Earth Planet. Sci. Letters* **495**, 192–201 (2018).
10. Affolter, S. et al. Central Europe temperature constrained by speleothem fluid inclusion water isotopes over the past 14,000 years. *Sci. Adv*. **5**: eaav3809, doi: 10.1126/sciadv.aav3809 (2019).
11. Jost, A. et al. High-resolution simulations of the last glacial maximum climate over Europe: a solution to discrepancies with continental palaeoclimatic reconstructions? *Climate Dyn.* **24**, 577–590 (2005).
12. Ramstein, G. et al. How cold was Europe at the Last Glacial Maximum? A synthesis of the progress achieved since the first PMIP model-data comparison. *Clim. Past* **3**, 331–339 (2007).
13. Strandberg, G. Brandefelt, J., Kjellström, E. & Smith, B. High-resolution regional simulation of last glacial maximum climate in Europe. *Tellus* **63A**, 107–125 (2011).
14. Rasmussen, S.U. et al. A stratigraphic framework for abrupt climatic changes during the Last Glacial period based on three synchronized Greenland ice-core records: refining and extending the INTIMATE event stratigraphy. *Quat. Sci. Rev.* **106**, 14–28 (2014).
15. Heiri, O. et al. Palaeoclimate records 60–8 ka in the Austrian and Swiss Alps and their forelands. *Quat. Sci. Rev.* **106**, 186–205 (2014).
16. Stojakowits, P. et al. Impact of climatic extremes on Alpine ecosystems during MIS 3. *Quat. Sci. Rev.* **239**, 106333 (2020).
17. Preusser, F., Graf, H. R., Keller, O., Krayss, E. & Schlüchter, C. Quaternary glaciation history of northern Switzerland. *Quat. Sci. J.* **60**, 282–305 (2011).
18. Spötl, C., Reimer, P.J., Starnberger, R. & Reimer, R. A new radiocarbon chronology of Baumkirchen, stratotype for the onset of the Upper Würmian in the Alps, *J. Quat. Sci.* **28**, 552–558 (2013).
19. Luetscher, M. et al. North Atlantic storm track changes during the Last Glacial Maximum recorded by Alpine speleothems. *Nature Commun.,* **6**:6344, doi.org/10.1038/ncomms7344 (2015).
20. Luetscher, M. &, Jeannin, P.Y. Temperature distribution in karst system: the role of air and water fluxes. *Terra Nova* **16**, 344–350 (2004).
21. Badino, G. Underground drainage systems and geothermal flux. *Acta Carsologica* **34**, 277–316 (2005).
22. Maréchal, J.C. & Perrochet, P. Theoretical relation between water flow rate in a vertical fracture and rock temperature in the surrounding massif. *Earth Planet. Sci. Letters* **194**, 213–219 (2001).
23. van Husen, D. *Die Ostalpen und ihr Vorland in der letzten Eiszeit (Würm)* Geol. Survey of Austria, Vienna (1987).
