# Supplementary material for: Increased autumn and winter precipitation during the Last Glacial Maximum in the European Alps
Source: Nat Commun. 2021 Mar 23;12:1839. doi: 10.1038/s41467-021-22090-7 (PMC7988052; doi:10.1038/s41467-021-22090-7)
Supplement: Supplementary file 2 — Descriptions of Additional Supplementary Files [file 41467_2021_22090_MOESM2_ESM.docx]

Descriptions of Additional Supplementary Files

**Supplementary Data 1**

**Description:** Stable isotope data of CCC from the different occurrences in the Obir caves.

**Supplementary Data 2**

**Description:** 230Th dating results of cryogenic calcite samples from the Obir caves. Sample numbers refer to those in Suppl. Figs. 2-5.
